# Increased expression of RUNX1 in clear cell renal cell carcinoma predicts poor prognosis

**DOI:** 10.7717/peerj.7854

**Published:** 2019-10-02

**Authors:** Yang Fu, Shanshan Sun, Xiaojun Man, Chuize Kong

**Affiliations:** 1 Departments of Urology, The First Hospital of China Medical University, Shenyang, Liaoning, China; 2 China Medical University, Shenyang, Liaoning, China; 3 Departments of Pharmacy, The First Hospital of China Medical University, Shenyang, Liaoning, China

**Keywords:** Clear cell renal cell carcinoma, RUNX1, Prognosis, GSEA, TCGA

## Abstract

**Background:**

Runt-related transcription factor 1 (RUNX1) was previously reported to play a dual role in promoting or suppressing tumorigenesis in various malignancies. A public dataset from The Cancer Genome Atlas (TCGA) was used to evaluate the role of RUNX1 in clear cell renal cell carcinoma (ccRCC).

**Methods:**

The Wilcoxon signed-rank test was used to compare the expression of RUNX1 in ccRCC tissues and normal tissues. The Wilcoxon signed-rank test and logistic regression were utilized to investigate the relationship between clinicopathological factors and RUNX1 expression. Additionally, we analysed the differences in prognosis between patients with high and low expression of RUNX1 via the Kaplan–Meier method and Cox regression. Gene set enrichment analysis (GSEA) was performed to explore the mechanisms of RUNX1 in ccRCC.

**Results:**

The expression of RUNX1 in ccRCC tissues was significantly higher than that in normal tissues. High expression of RUNX1 was significantly associated with gender (*p* = 0.003), clinical stage (*p* < 0.001), tissue infiltration (*p* < 0.001), lymph node metastasis (*p* = 0.037) and histological grade (*p* < 0.001). Logistic regression analysis showed that high RUNX1 expression was significantly correlated with gender (OR = 1.71 for male vs. female, *p* = 0.004), histological grade (OR = 11.61 for grade IV vs. I, *p* < 0.001), clinical stage (OR = 1.55 for stage III/IV vs. I/II, *p* = 0.014) and tissue infiltration (OR = 1.54 for positive vs. negative, *p* = 0.018). Kaplan–Meier survival curves revealed that the prognosis of patients with ccRCC with high RUNX1 expression was worse than that of patients with ccRCC with low RUNX1 expression (*p* < 0.001). Univariate Cox regression analysis showed that high RUNX1 expression was strongly correlated with poor prognosis (HR = 1.60, 95% CI [1.31–1.97], *p* < 0.001). In addition, high expression of RUNX1 was an independent prognostic factor for poor overall survival (OS), with an HR of 1.50 (95% CI [1.20–1.87], *p* < 0.001) in multivariate Cox analysis. GSEA showed that the apoptosis, B cell receptor signalling pathway, calcium signalling pathway, chemokine signalling pathway, JAK/STAT signalling pathway, MAPK signalling pathway, p53 signalling pathway, pathways in cancer, T cell receptor signalling pathway, Toll-like receptor signalling pathway, VEGF signalling pathway, and Wnt signalling pathway were significantly enriched in the RUNX1 high-expression phenotype. In conclusion, RUNX1 can be used as a novel prognostic factor and therapeutic target in ccRCC.

## Introduction

Renal cell carcinoma (RCC) accounts for nearly 85% of primary renal carcinomas, and in the United States, approximately 63,000 new cases and nearly 14,000 deaths occur each year (Garfield & LaGrange, 2019). The most common pathological type of RCC is clear cell RCC (ccRCC), which accounts for approximately 70–75% of cases (Shuch et al., 2015). However, 30% of patients with ccRCC are diagnosed with advanced cancer (Karakiewicz et al., 2007). The optimal treatment for localized ccRCC is radical resection, but the therapies available for advanced cancer with metastasis are not very effective. Currently, targeted therapy has become a promising strategy. Therefore, the identification of hub genes associated with ccRCC is important for targeted therapy.

Runt-related transcription factor 1 (RUNX1) is widely considered the master regulator of haematopoiesis that mediates endothelial-to-haematopoietic transition (EHT) and the homeostasis of haematopoietic stem and progenitor cells (HSPCs) (Yzaguirre, De Bruijn & Speck, 2017; Yzaguirre et al., 2018; Cheng et al., 2017). The results of increasing studies imply that RUNX1 has contrasting effects of promoting or suppressing tumorigenesis in various malignancies. For example, upregulation of RUNX1 led to the increased expression of SOS1 and phosphorylation of ErbB2/HER2, subsequently promoting the proliferation of gastric cancer cells (Mitsuda et al., 2018). In the ER+ subtype of breast cancer, RUNX1 has been implicated as a tumour suppressor through the stabilization of Axis inhibition protein 1 (AXIN1) expression, but high RUNX1 expression correlates with poor prognosis in triple-negative breast cancers (Mercado-Matos, Matthew-Onabanjo & Shaw, 2017; Chimge et al., 2016). Additionally, loss of RUNX1 resulted in enhanced proliferation, migration, and invasion in lung cancer (Ramsey et al., 2018). Recently, the literature in ccRCC reported the upregulation of RUNX1- RUNX1 partner transcriptional co-repressor 1 (RUNX1-RUNX1T1) gene signatures, the fusion transcript between the RUNX1 gene and the RUNX1T1 locus because of t(8;21)(q22;q22) (Metzeler & Bloomfield, 2017), which was revealed by RNA sequencing (Xiong et al., 2014). However, the relationship between RUNX1 and the prognosis of ccRCC is unknown.

Therefore, we assessed RUNX1 expression in ccRCC and evaluated the correlation between RUNX1 expression and the prognosis of ccRCC using the public dataset from The Cancer Genome Atlas (TCGA). GSEA was performed to explore the mechanisms of RUNX1 in ccRCC.

## Materials and Methods

### Basic patient information

The mRNA expression data and the corresponding clinical data of ccRCC patients were obtained from the official website of The Cancer Genome Atlas (TCGA). After excluding normal sample data, boxplots were used to analyse the relationship between RUNX1 expression and various clinical variables. Ultimately, we included 537 ccRCC patients with RUNX1 mRNA expression data and clinical data (Table 1).

**Table 1:** Characteristics of clear cell renal cell carcinoma patients in the TCGA database.

Clinical characteristics		Total (*n* = 537)	%
Age at diagnosis (years)		61 (26–90)	
Gender	Female	191	35.6
	Male	346	64.4
Histological grade	I	14	2.6
	II	230	43.5
	III	207	39.2
	IV	78	14.7
Stage	I	269	50.3
	II	57	10.7
	III	125	23.4
	IV	83	15.6
Tissue infiltration^a^	Negative	344	64.1
	Positive	193	35.9
Lymph node metastasis^b^	Negative	240	93.3
	Positive	17	6.7
Distant metastasis^c^	Negative	426	84.3
	Positive	79	15.7

**Notes.**

a Positive indicates tissue infiltration and negative indicates no tissue infiltration.

b Positive indicates lymph node metastasis and negative indicates no lymph node metastasis.

c Positive indicates distant metastasis and negative indicates no distant metastasis.

### Gene set enrichment analysis (GSEA)

GSEA was utilized to explore the mechanisms of RUNX1 in ccRCC. In this study, the “c2.cp.kegg.v6.2.symbols.gmt” gene sets from the Molecular Signatures Database (MSigDB) were analysed using GSEA 3.0 software. To obtain normalized enrichment scores (NESs), the nominal *p*-value and false discovery rate (FDR) *q*-value were determined, the number of gene set permutations for each analysis was set at 1,000, and the phenotype label was the expression level of RUNX1. Gene sets with a nominal *p*-value of <0.05 and an FDR *q*-value of <0.25 were considered significantly enriched gene sets.

### Statistical analysis

The median value of RUNX1 expression was considered as the cut-off value. The Wilcoxon signed-rank test was used to compare the expression of RUNX1 in ccRCC tissues with that in normal tissues. The Wilcoxon signed-rank test and logistic regression were utilized to investigate the relationship between clinicopathological factors and RUNX1. The correlation between RUNX1 expression and overall survival (OS) was estimated using Kaplan–Meier survival curves. Univariate Cox regression analysis was used to calculate the association of clinicopathological characteristics and RUNX1 expression with OS, and multivariate Cox analysis was carried out for further analysis. All statistical analyses were performed using R 3.5.3 software (R Core Team, 2018).

## Results

### Patient characteristics

A total of 537 primary tumours with both gene expression and clinical data were downloaded from TCGA ccRCC data in May 2019. In our study cohort, the median age was 63 years (range, 26–90 years). Histological grades I, II, III, and IV accounted for 2.6%, 43.5%, 39.2%, and 14.7% of the cases, respectively. Stages I, II, III and IV accounted for 50.3%, 10.7%, 23.4% and 15.6% of the cases, respectively. A total of 193 of the 537 (35.9%) cases had tissue infiltration. A total of 17 of 257 (6.7%) cases had lymph node metastasis, and 79 of 505 (6.7%) cases had distant metastasis (Table 1).

### RUNX1 was highly expressed in ccRCC tissues

**Figure 1:**
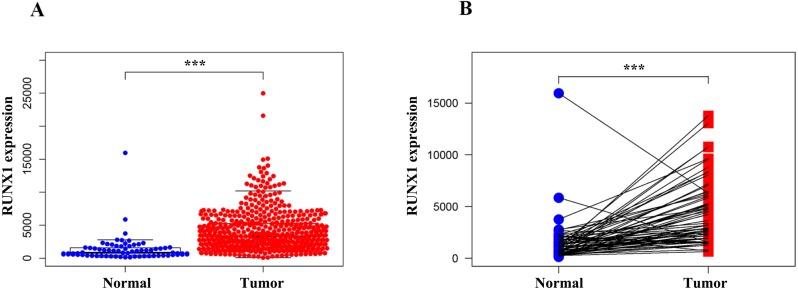
Comparison of RUNX1 expression between ccRCC tissues and normal tissues. The Wilcoxon signed-rank test was used to compare the mRNA expression of RUNX1 in ccRCC tissues and normal tissues obtained from the TCGA database. (A) The expression of RUNX1 in ccRCC tissues (*n* = 539) was significantly higher than that in normal tissues (*n* = 72). (B) Compared with 72 paired normal tissues, ccRCC tissues exhibited obvious overexpression of RUNX1. RUNX1, Runt-related transcription factor 1; ccRCC, clear cell renal cell carcinoma; TCGA, The Cancer Genome Atlas ; *, *p* < 0.05 ; **, *p* < 0.01; ***, *p* < 0.001.

We compared the expression of RUNX1 in 539 ccRCC tissues and 72 normal tissues using the Wilcoxon signed-rank test. The expression of RUNX1 in ccRCC tissues was significantly higher than that in normal tissues (*p* < 0.001) (Fig. 1A). In addition, RUNX1 was also obviously overexpressed in the ccRCC tissues relative to its expression in the 72 paired normal tissues (*p* < 0.001) (Fig. 1B).

### The expression of RUNX1 and its association with clinicopathological variables

**Figure 2:**
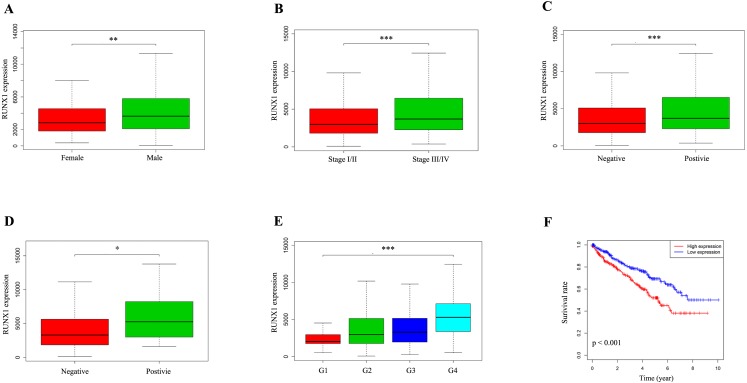
The expression of RUNX1 and its association with clinicopathologic variables. The median value of RUNX1 expression was considered as the cut-off value. The Wilcoxon signed-rank test was utilized to investigate the relationship between clinicopathological factors and the expression of RUNX1 in 537 ccRCC tissues. The correlation between RUNX1 expression and overall survival (OS) was estimated using Kaplan–Meier survival curves. (A) Gender, (B) clinical stage, (C) tissue infiltration, (D) lymph node metastasis, (E) histological grade and (F) Kaplan–Meier survival curves showed that the prognosis of patients with ccRCC with high RUNX1 expression was worse than that of patients with ccRCC with low RUNX1 expression. In (C), positive indicates tissue infiltration and negative indicates no tissue infiltration. In (D), positive indicates lymph node metastasis and negative indicates no lymph node metastasis. RUNX1, Runt-related transcription factor 1; ccRCC, clear cell renal cell carcinoma; *, *p* < 0.05; **, *p* < 0.01; ***, *p* < 0.001.

We used the Wilcoxon signed-rank test to analyse 537 ccRCC samples from the TCGA database with both RUNX1 expression data and clinical data. The median value of RUNX1 expression was considered as the cut-off value. As shown in Figs. 2A–2E, high expression of RUNX1 was significantly associated with gender (*p* = 0.003), clinical stage (*p* < 0.001), tissue infiltration (*p* < 0.001), lymph node metastasis (*p* = 0.037) and histological grade (*p* < 0.001). The logistic regression analysis results showed that high RUNX1 expression was significantly correlated with gender (odds ratio [OR] = 1.71 for male vs. female, *p* = 0.004), histologic grade (OR = 11.61 for grade IV vs. I, *p* < 0.001), stage (OR = 1.55 for stage III/IV vs. I/II, *p* = 0.014) and tissue infiltration (OR = 1.54 for positive vs. negative, *p* = 0.018) (Table 2).

**Table 2:** Association of RUNX1 expression^a^ with clinicopathological characteristics (logistic regression analysis).

Clinical characteristics	Total (N)	Odds ratio for RUNX1 expression	*p*-value
Age (continuous)	537	0.99 (0.98–1.01)	0.659
Gender (male vs. female)	537	1.71 (1.19–2.45)	0.004
Grade (IV vs. I)	92	11.61 (3.22–55.67)	0.000
Stage (III/IV vs. I/II)	527	1.55 (1.09–2.21)	0.014
Distant metastasis (positive vs. negative)^b^	505	1.36 (0.84–2.21)	0.219
Tissue infiltration (positive vs. negative)^c^	537	1.54 (1.07–2.19)	0.018
Lymph node metastasis (positive vs. negative)^d^	257	1.73 (0.63–5.24)	0.299

**Notes.**

a Categorical dependent variable, greater or less than the median expression level.

b Positive indicates distant metastasis and negative indicates no distant metastasis.

c Positive indicates tissue infiltration and negative indicates no tissue infiltration.

d Positive indicates lymph node metastasis and negative indicates no lymph node metastasis.

### Survival outcomes and Cox regression analyses

Kaplan–Meier survival curves revealed that the prognosis of patients with ccRCC with high RUNX1 expression was worse than that of patients with ccRCC with low RUNX1 expression (*p* < 0.001) (Fig. 2F). The univariate Cox regression analysis results showed that high RUNX1 expression was strongly correlated with poor prognosis (hazard ratio [HR] = 1.60, 95% confidence interval CI [1.31–1.97], *p* < 0.001; Table 3A). Other clinical variables, such as age, grade, stage, distant metastasis, tissue infiltration and lymph node metastasis, were found to have an impact on OS (all *p* values <0.05). Moreover, in multivariate analysis, high expression of RUNX1 (HR =1.50, 95% CI [1.20–1.87], *p* < 0.001), age (HR =1.03, 95% CI [1.01–1.05], *p* = 0.002) and distant metastasis (HR =2.42, 95% CI [1.14–5.16], *p* = 0.022) still implied a poor prognosis (Table 3A). These results indicate that the expression of RUNX1 is an independent factor and that increased RUNX1 levels are associated with poor OS.

**Table 3:** Association with overall survival and clinicopathological characteristics in patients from the TCGA database using Cox regression. (A) Univariate Cox regression (B) Multivariate Cox regression.

Clinicopathological variables	Hazard ratio (95% CI)	*p*-value
(**A**)		
Age (continuous)	1.02 (1.00–1.04)	0.012
Gender (male vs. female)	1.01 (0.67–1.54)	0.951
RUNX1 expression (high vs. low)	1.60 (1.31–1.97)	0.000
Grade	2.24 (1.68–2.99)	0.000
Stage	1.86 (1.54–2.25)	0.000
Distant metastasis (positive vs. negative)^a^	4.07 (2.63–6.30)	0.000
Tissue infiltration (positive vs. negative)^b^	3.31 (2.17–5.06)	0.000
Lymph node metastasis (positive vs. negative)^c^	2.93 (1.52–5.67)	0.001
(**B**)		
Age (continuous)	1.03 (1.01–1.05)	0.002
RUNX1 expression (high vs. low)	1.50 (1.20–1.87)	0.000
Distant metastasis (positive vs. negative)	2.42 (1.14–5.16)	0.022

**Notes.**

a Positive indicates distant metastasis and negative indicates no distant metastasis.

b Positive indicates tissue infiltration and negative indicates no tissue infiltration.

c Positive indicates lymph node metastasis and negative indicates no lymph node metastasis.

### GSEA

**Figure 3:**
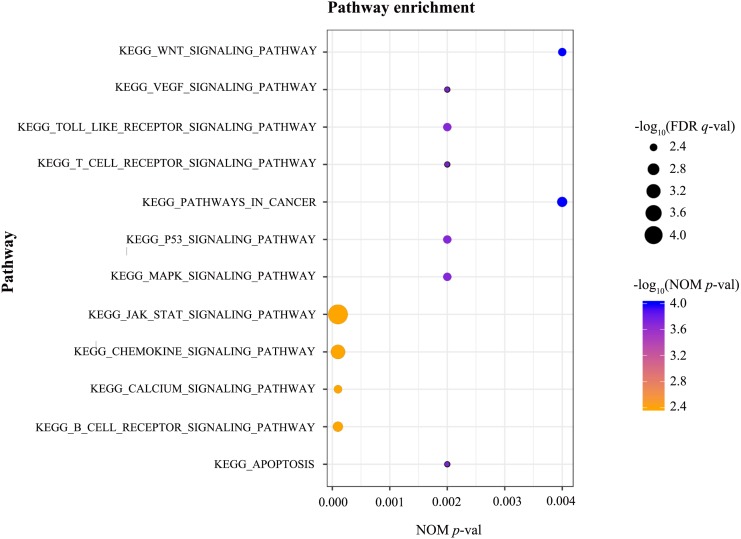
GSEA. GSEA was conducted to identify the differentially activated signalling pathways in the high RUNX1 expression ccRCC data sets. GESA showed that the apoptosis, the B cell receptor signalling pathway, the Calcium signalling pathway, the chemokine signalling pathway, the JAK/STAT signalling pathway, the MAPK signalling pathway, the p53 signalling pathway, pathways in cancer, the T cell receptor signalling pathway, the Toll-like receptor signalling pathway, the VEGF signalling pathway, and the Wnt signalling pathway were significantly enriched in the RUNX1 high-expression phenotype. GSEA, gene set enrichment analysis; RUNX1, Runt-related transcription factor 1; ccRCC, clear cell renal cell carcinoma; NOM *p*-val, nominal *p*-value ; FDR *q*-val, false discovery rate *q*-value.

GSEA was conducted to identify the differentially activated signalling pathways in the high RUNX1 expression ccRCC data sets. The results shown in Table 4 and Fig. 3 revealed the most significant signalling pathways enriched in the RUNX1 high-expression phenotype.

**Table 4:** Gene sets enriched in the high-expression phenotype.

Gene set name	NES	NOM *p*-val	FDR *q*-val
KEGG_APOPTOSIS	1.970	0.002	0.005
KEGG_B_CELL_RECEPTOR_SIGNALING_PATHWAY	2.069	0.000	0.003
KEGG_CALCIUM_SIGNALING_PATHWAY	2.021	0.000	0.004
KEGG_CHEMOKINE_SIGNALING_PATHWAY	2.145	0.000	0.001
KEGG_JAK_STAT_SIGNALING_PATHWAY	2.242	0.000	0.000
KEGG_MAPK_SIGNALING_PATHWAY	1.997	0.002	0.004
KEGG_P53_SIGNALING_PATHWAY	2.022	0.002	0.004
KEGG_PATHWAYS_IN_CANCER	2.075	0.004	0.003
KEGG_T_CELL_RECEPTOR_SIGNALING_PATHWAY	1.973	0.002	0.005
KEGG_TOLL_LIKE_RECEPTOR_SIGNALING_PATHWAY	2.007	0.002	0.004
KEGG_VEGF_SIGNALING_PATHWAY	1.966	0.002	0.005
KEGG_WNT_SIGNALING_PATHWAY	2.009	0.004	0.004

**Notes.**

NES normalized enrichment score NOM nominal; FDR false discovery rate

Gene sets with NOM *p*-val <0.05 and FDR *q*-val <0.25 were considered significant.

## Discussion

RUNX1, located on chromosome 21q22.3, consists of 12 exons with two distinct promoters and modulates the transcription of its target genes by affecting the DNA binding capacity (De Braekeleer et al., 2011; Mitsuda et al., 2018; Liu et al., 2019). Researchers have demonstrated differential expression of RUNX1 in several cancers, and deregulation of RUNX1 correlates with tumour progression, which could ultimately lead to its dual role. For example, the expression of RUNX1 in hepatocellular carcinoma tissues is much lower than that in adjacent normal tissues and adjacent cirrhotic tissues (Miyagawa et al., 2006). In addition, RUNX1 is overexpressed in non-small cell lung cancer (NSCLC) and promotes the invasion of NSCLC cells (Wang et al., 2015). RUNX1 was also confirmed to be upregulated in endometrial cancer and related to myometrial invasion (Planaguma et al., 2004). Knockdown of RUNX1 inhibited proliferation and apoptosis induced by Leucine-rich-alpha-2-glycoprotein 1 (LRG1) in colorectal cancer cells (Zhou et al., 2017). RUNX1 has been shown to be androgen-dependent, and loss of RUNX1 may contribute to prostate cancer progression (Takayama et al., 2015). Additionally, RUNX1 suppress the growth of several breast cancer cell lines through the repression of cancer stem cell activity and inhibition of zinc finger E-Box binding protein 1 (ZEB1) expression (Hong et al., 2018). However, the effect of RUNX1 expression on the prognosis of RCC, which is the focus of the current study, remains unclear.

Here, we confirmed that the RUNX1 high-expression phenotype was closely correlated with gender, high histological grade, advanced clinical stage, tissue infiltration and poor prognosis. GSEA was performed to further investigate the biological functions of RUNX1 in ccRCC. GESA is one of the most commonly used methods for pathway analysis in tumours. The results of GESA are based on gene sets rather than a single gene, making these results more reliable and flexible than those obtained using traditional methods (Subramanian et al., 2005). However, the approach of GESA, functional class scoring (FCS), has several limitations. FCS analyses each pathway independently and treats differently significant genes equally, which may overlook the biological significance of genes within these pathways and their complex interactions with each other (Khatri, Sirota & Butte, 2012). Additionally, it is difficult to set the appropriate threshold for determining gene sets for pathways with insufficient annotation information, which may lead to the loss of some less significant but still critical genes, resulting in reduced detection sensitivity. GESA showed that apoptosis, the B cell receptor signalling pathway, the calcium signalling pathway, the chemokine signalling pathway, the JAK/STAT signalling pathway, the MAPK signalling pathway, the p53 signalling pathway, pathways in cancer, the T cell receptor signalling pathway, the Toll like receptor signalling pathway, the VEGF signalling pathway, and the Wnt signalling pathway were significantly enriched in the RUNX1 high-expression phenotype. Some of these pathways have been reported in the literature related to other cancers. The distal P1-Runx1 promoter may be important in the onset or progression of leukaemia via Wnt/beta-catenin signalling (Medina et al., 2016). RUNX1 gene silencing induces p53-promoted core binding factor-beta (CBFB) expression, and upregulated CBFB stabilizes RUNX1 expression in acute myeloid leukaemia cells, resulting in a compensatory RUNX1-p53-CBFB feedback loop (Morita et al., 2017). Moreover, IL-1*β* induces RUNX1 expression through the MAPK signalling pathway and promotes migration, invasion, and angiogenesis in glioblastoma (Sangpairoj et al., 2017). However, the association of RUNX1 with the ccRCC signalling pathways has not been studied. Our findings suggest that RUNX1 can be used as a novel biomarker for predicting poor prognosis and as a therapeutic target in ccRCC, but the underlying mechanisms need to be further elucidated.

Although the current study elaborated on the role of RUNX1 in ccRCC, it still has some limitations. First, the number of tumour tissues in the TCGA database was significantly higher than the number of normal tissues used as a control. Second, specific details, such as the use of drugs, surgical treatment and surgical details, are lacking, and these factors are important for patient prognosis. Finally, the protein levels and direct mechanisms of RUNX1 in ccRCC could not be clearly assessed from the TCGA database. Therefore, further studies on RUNX1 in ccRCC are needed.

## Conclusions

The expression of RUNX1 may be associated with poor survival in ccRCC, and the apoptosis, the B cell receptor signalling pathway, the calcium signalling pathway, the chemokine signalling pathway, the JAK/STAT signalling pathway, the MAPK signalling pathway, the p53 signalling pathway, pathways in cancer, the T cell receptor signalling pathway, the Toll-like receptor signalling pathway, the VEGF signalling pathway, and the Wnt signalling pathway may be significantly correlated with RUNX1 expression in ccRCC. The biological role of RUNX1 in ccRCC should be further assessed in future studies.

##  Supplemental Information

10.7717/peerj.7854/supp-1 Supplemental Information 1 Raw data

## Additional Information and Declarations

### Data Availability

The following information was supplied regarding data availability: The raw data are available in the Supplemental File.
