# Supplementary material for: Increased expression of RUNX1 in clear cell renal cell carcinoma predicts poor prognosis
Source: PeerJ. 2019 Oct 2;7:e7854. doi: 10.7717/peerj.7854 (PMC6778431; doi:10.7717/peerj.7854)
Supplement: Supplemental Information 1 [file peerj-07-7854-s001.zip › Description.docx]

1-2 are used to compare the expression of RUNX1 in ccRCC tissues and normal tissues.

3-9 are used to investigate the relationship between clinicopathological factors and RUNX1 expression.

10 is the raw data of the Cox regression.

11 is the raw data of the Kaplan–Meier method.

12 is the raw data of the GESA.
